# The Relationship Between Emotion Processing Assessed by an Affect Rating Task and Depression Symptoms Following the Accelerated Sequential Dorsolateral–Dorsomedial Prefrontal rTMS Treatment

**DOI:** 10.3390/bs16020178

**Published:** 2026-01-26

**Authors:** Ruiqin Chen, Zerun Dong, Ruijie Geng, Haibin Li, Yuan Wang, Yuanyuan Li, Qiong Ding, Yingying Zhang, Xuechen Ding, Jingjing Huang, Hui Zhao, Wenjuan Liu, Valerie Voon, Yi-Jie Zhao

**Affiliations:** 1 Clinical Research Center for Mental Disorders, Shanghai Pudong New Area Mental Health Center, School of Medicine, Tongji University, Shanghai 200124, China; 2 Institute of Science and Technology for Brain-Inspired Intelligence, Fudan University, Shanghai 200433, China; 3 Department of Psychology, Shanghai Normal University, Shanghai 200234, China; 4 Department of Psychological Medicine, Zhongshan Hospital, Fudan University, Shanghai 200032, China; 5 Department of Psychiatry, University of Cambridge, Cambridge CB2 0QQ, UK; 6 Department of Psychiatry, Shanghai United Family Pudong Hospital, Shanghai 201206, China

**Keywords:** major depressive disorder, accelerated rTMS, affect, emotion, Affect Rating Task (ART), predictor

## Abstract

Background: Emotion processing is critical in the neuropathology of major depressive disorder (MDD), while its relationship with clinical treatment remains unclear. This study aims to indicate the associations between emotion processing and treatment effects following a sequential dual-site accelerated repetitive transcranial magnetic stimulation (rTMS) protocol. Methods: MDD patients were recruited to receive rTMS treatment with four sessions per day for four consecutive days, with stimulation sequentially delivered to the left dorsolateral prefrontal cortex (dlPFC) and the dorsomedial prefrontal cortex (dmPFC). Symptoms were assessed at baseline, end of treatment, and week 4 using the Montgomery–Åsberg Depression Rating Scale (MADRS), Snaith-Hamilton Pleasure Scale (SHAPS), and Fatigue Severity Scale (FSS). Emotional valence and arousal were evaluated with the Affect Rating Task (ART). Results: A total of 51 participants completed the clinical assessments and ART, with two excluded due to missing baseline data in the SHAPS and FSS. The linear mixed-effects models revealed significant improvement in depressive (*p* < 0.001, d = −0.343) and fatigue symptoms (*p* = 0.010, d = −0.572) following rTMS treatment. Neutral valence was correlated with MADRS scores at baseline (R^2^ = 0.096, *p* = 0.027). In addition, changes in arousal for positive images (*p* = 0.047, adjusted R^2^ = 0.097) and neutral images (*p* = 0.019, adjusted R^2^ = 0.160) at treatment end were significantly correlated with MADRS improvement at week 4. Conclusions: Our study highlights the association between changes in emotional arousal and improvement in MDD following accelerated dlPFC-dmPFC dual-site rTMS treatment.

## 1. Introduction

Major depressive disorder (MDD) represents a critical global mental health challenge and is characterized by significant neuropsychological dysfunction (Wang et al., 2025). Emerging neurobiological evidence suggests a profound correlation between cognitive processing, particularly emotion processing, and clinical manifestation of depressive symptoms, indicating that impaired emotion processing may serve as a core neuropsychological mechanism underlying MDD progression (Gollan et al., 2008; Millan et al., 2012; Ochsner & Gross, 2005; Sterzer et al., 2011; Wen et al., 2023). However, the relationship between emotion processing and anti-depressive treatment effects remains unclear.

Dysfunctional emotion processing serves as a core factor in depression, with its abnormalities functioning as fundamental symptomatic markers. Evidence has revealed that MDD patients commonly exhibit impaired emotion recognition, indicated by reduced accuracy in the identification of happy facial expressions in severe individuals (Dalili et al., 2014; Krause et al., 2021). Critically, it is suggested that depressed patients exhibit a negative bias and blunted positivity in emotion processing (Rizeq, 2024). Neuropsychological models further posit that antidepressant treatments may act through the correction of negative cognitive bias (Ma et al., 2025; Roiser et al., 2011), while the efficacy of neuromodulation approaches like repetitive Transcranial Magnetic Stimulation (rTMS) in improving emotion processing remains incompletely characterized. Systematic review shows that rTMS has positive effects on cognitive control of emotion, but most studies are conducted on healthy individuals (Lantrip et al., 2017; Nejati et al., 2022). Research in depression patients reports that rTMS enhances performance in the affective go/no-go task and improves inhibitory processing of negative information assessed by the negative affective priming task (Bermpohl et al., 2006; Leyman et al., 2011). Overall, it is believed that emotion processing plays a critical role in disease maintenance, while its relationship with treatment and prognosis requires further investigation.

Impairment in emotion processing in MDD is related to large-scale network dysfunction, involving affective, reward, and attentional networks, with high heterogeneity (Drysdale et al., 2017). Converging evidence has shown that MDD patients exhibit weaker activation in the prefrontal cortex during emotion processing, particularly in the dorsolateral prefrontal cortex (dlPFC) (Trettin et al., 2022), which is used as an rTMS target approved by the FDA for MDD treatment. In addition, meta-analysis results indicate that patients with depression exhibit reduced dorsomedial prefrontal cortex (dmPFC) gray matter volume (Bora et al., 2012), and importantly, rTMS targeting the dmPFC demonstrates therapeutic efficacy for depressive symptoms and anhedonia (Downar et al., 2014). Furthermore, neuroimaging evidence implicates hyperconnectivity within the Default Mode Network (DMN) in depression, particularly involving the bilateral dmPFC, which is related to depression-relevant cognitive functions, including emotion processing and self-reflection (Downar & Daskalakis, 2013; Greicius et al., 2007; Sheline et al., 2010; Sun et al., 2023). Thus, it is suggested that functional alterations of dmPFC may constitute a key mechanistic pathway.

Based on evidence of abnormal activity in the brain networks, our group developed a sequential rTMS protocol targeting the dlPFC followed by the dmPFC and demonstrated rapid and sustained antidepressant effects (Zhao et al., 2025). In the present study, we further investigated the emotion processing changes caused by this treatment. We employed an Affective Rating Task (ART) to test the emotional valence and arousal to different types of images. Clinical assessments focused on depression symptoms, anhedonia, and fatigue symptoms. We hypothesized that the changes in emotion processing as a function of our dual-site rTMS treatment may relate to the therapeutic effects. The primary objective of this exploratory research was to investigate the influence of our dual-site rTMS on emotion processing and to explore whether changes in emotion processing could predict the outcome of the treatment.

## 2. Materials and Methods

### 2.1. Participants

A total of 52 participants meeting the Diagnostic and Statistical Manual of Mental Disorders, Fifth Edition (DSM-5) criteria for depression finished the primary clinical trial. This sample size meets the statistical power requirements for the clinical trial. Details about power analysis, inclusion, and exclusion criteria can be found in Appendix A and our previous related publication (Zhao et al., 2025). Participants were randomly assigned to either the active group receiving real stimulation or the sham group receiving sham stimulation. One participant failed to finish the ART test. As a result, data from 51 participants were included in the final analysis (26 in the active group and 25 in the sham group). For the analyses of SHAPS and FSS, the sample size was 49 due to missing baseline data (25 in the active group and 24 in the sham group).

### 2.2. Randomization and Blinding of Treatment

Using computer-generated randomization, participants were allocated 1:1 to active or sham rTMS groups. Both assessors and participants remained blind to treatment conditions. Treatment administrators, who were unblinded and distinct from assessors, delivered the interventions. Immediately preceding unblinding, both participants and assessors systematically documented their allocation conjectures. Details of the blinding procedure and results are reported in the previous publication (Zhao et al., 2025).

### 2.3. Transcranial Magnetic Stimulation

TMS was delivered using a pulsed magnetic stimulation device (M-100 Ultimate, Shenzhen Yingchi Technology Co., Ltd., Shenzhen, China) with a liquid-cooled flat figure-8 coil (77 mm diameter, model BY90A) targeting both sites. Resting motor threshold (RMT) was determined over the left motor cortex as the minimum intensity required to elicit motor evoked potentials (MEPs) > 0.05 mV in at least 5 out of 10 trials, recorded via electrodes on the right abductor pollicis brevis muscle. Both dlPFC and dmPFC targets were stimulated at 100% individual RMT. The left dlPFC was localized 5 cm anterior to the M1, while the dmPFC coordinates corresponded to 25% of the nasion-inion midline distance (Miron et al., 2019). Sham stimulation was administered with the coil tilted 90° perpendicular to the skull.

A 20 Hz rTMS sequence was used for both targets, in which rTMS was on for 2 s and off for 4 s and repeated 30 times, resulting in a 3 min sequence with 1200 pulses in total (Miron et al., 2019). One treatment session consisted of one 3 min left dlPFC stimulation followed by one 3 min dmPFC stimulation with a 5 min interval. The inter-session interval was at least 50 min (Cole et al., 2022). Sixteen rTMS sessions were administered for 4 consecutive days, with 4 sessions per day. Each participant received 19,200 pulses over each stimulation site.

### 2.4. Assessment

The primary outcome measure was the Montgomery–Åsberg Depression Rating Scale (MADRS) rated by a psychiatrist. In addition, the self-rated Snaith-Hamilton Pleasure Scale (SHAPS) (Trøstheim et al., 2020) and Fatigue Severity Scale (FSS) (Hewlett et al., 2011) were also used to assess anhedonia and fatigue. Lower scores in MADRS and FSS indicated milder symptoms, whereas higher scores reflected improved anhedonia in SHAPS. All assessments were conducted before treatment (baseline), on the day after the last session of treatment (treatment end), and four weeks after treatment (week-4).

### 2.5. Affect Rating Task (ART)

**Figure 1:**
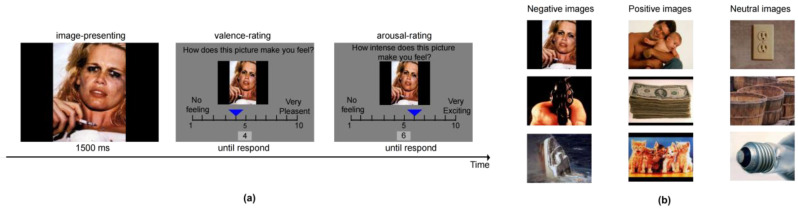
Affect Rating Task (ART). (**a**) Task procedure. Participants viewed each image for 1500 ms and then completed two untimed ratings using the “left” and “right” keys to move the indicator and assess valence and arousal, and the next image appeared only after both ratings were finished. (**b**) Examples of images used in the ART. The first column shows negative images, the second shows positive images, and the third shows neutral images.

The ART was used to assess the patient’s ability to experience emotions, which involved 6 measurements taken during the treatment period: at baseline, after each treatment day (4 days in total), and at week 4. Stimuli were 120 pictures selected from the International Affective Picture System (IAPS) (Lang et al., 2008), consisting of 40 positive (mean valence = 7.5 ± 0.16, mean arousal = 5.15 ± 0.98), 40 neutral (mean valence = 4.99 ± 0.11, mean arousal = 3.52 ± 1.00), and 40 negative (mean valence = 2.49 ± 0.17, mean arousal = 5.69 ± 0.65) pictures. Each measurement consisted of 30 trials, including 10 positive, 10 neutral, and 10 negative images randomly selected from the 120-picture pool. Participants were required to rate both valence and arousal of these images (Figure 1). Each image was displayed for 1500 ms, followed by an untimed rating phase. The next image appeared only after participants completed two ratings. Participants used the “left” or “right” key to move the blue indicator to rate images, and the corresponding score was displayed at the bottom of the screen simultaneously. For each rating, the initial score was randomly set between 4 and 7. Lower valence indicated that the patient perceived the image as more negative, while lower arousal signified that the emotional activation triggered by the image was relatively low.

### 2.6. Statistical Analysis

The Statistical Packages for Social Sciences (SPSS) 27.0 and R (version 4.3.3) software were used for statistical analysis.

Comparisons between continuous variables in demographic and clinical data were conducted using independent-sample *t*-tests in SPSS, while comparisons between categorical variables were performed using Chi-square tests. Pearson correlation analysis in SPSS was used to assess the correlation between baseline ART ratings and the baseline scores of MADRS, SHAPS, and FSS, with missing baseline data excluded.

Given the repeated measurements in ART and these clinical scales, prior to analysis, missing data were examined using Little’s MCAR test in SPSS, which indicated that data were missing completely at random (*χ*^2^_(164)_ = 152.432, *p* = 0.732). Therefore, mixed-effects modeling with full information maximum likelihood was considered appropriate.

We applied linear mixed-effects models (LMM) using the *lme4* and *lmerTest* packages, and linear regression models for longitudinal analysis using the base function *lm()* in R. The LMM provided a flexible framework to capture individual trajectories over time, whereas the linear regression models in SPSS were used to examine associations between the rates of change in key variables, after which multiple linear regression models (MLR) were conducted to test the associations. Missing values were not imputed in these models, since the LMM can handle missing data under the missing-at-random assumption, while the linear regression analysis only included participants with complete data on the relevant variables. The models controlled for participants’ age and sex as covariates. All *p*-values were two-sided and adjusted for multiple comparisons using False Discovery Rate (FDR) correction.

Model assumptions were evaluated both visually and statistically. For the MLR models, multicollinearity (all VIF < 5), normality (residual plots and Shapiro–Wilk tests), homoscedasticity (Breusch-Pagan tests), and influential observations (Cook’s distance) were examined. Bootstrapping procedures were applied for robust estimation. For the LMM, diagnostic tests from the *DHARMa* package were used, including uniformity (Kolmogorov–Smirnov test), dispersion, and outlier diagnostics based on simulated scaled residuals. All models showed satisfactory fit, with no substantial violations of distributional assumptions or convergence issues.

## 3. Results

### 3.1. Demographic and Clinical Data

Demographic results showed that active and sham groups were not significantly different in sex, age, education level, disease duration, episodes, baseline MADRS (Table 1), or medication, which indicated that the active and sham groups were comparable at baseline in demographic variables, reducing concern about baseline confounding. Medication use was kept stable during the treatment period, thereby serving as a design-level control for potential pharmacological confounds.

No participant quit in the middle of treatment because of discomfort, indicating high tolerability of our protocol. No severe adverse events occurred during the trial. The most common adverse event was discomfort at the stimulation site, which was self-resolved. Details about adverse events were reported in our previous publication (Zhao et al., 2025).

To confirm the clinical efficacy of our rTMS treatment on the subset of participants relative to our original clinical trial paper (Zhao et al., 2025), we first conducted an LMM on the clinical data with three timepoints (baseline, treatment end, week 4) and two groups (active vs. sham). For MADRS, the main effects of time (*F*_(2,98)_ = 49.600, *p* < 0.001, η^2^ = 0.503) and group (*F*_(1,47)_ = 5.511, *p* = 0.023, *η*^2^ = 0.001) were significant. Significant interactions between time and group were also observed (*F*_(2,98)_ = 8.248, *p* < 0.001, *η*^2^ = 0.144). Post hoc tests confirmed that participants in the active group showed lower scores than the sham group both at the treatment end (*β* = −4.254, SE = 1.813, *t*_(119)_ = −2.342, *p* = 0.021, 95% CI [−7.84, −0.65], *d* = −0.214) and week 4 (*β* = −6.783, SE = 1.813, *t*_(119)_ = −3.742, *p* < 0.001, 95% CI [−10.38, −3.19], *d* = −0.343), but scores of the two groups exhibit no difference at baseline (*p* = 0.373). Thus, after rTMS treatment, depression symptoms in the active group were reduced more markedly compared with the sham group.

Response (≥50% reduction in MADRS) rates at week 4 were 46.15% in the active group and 12.00% in the sham group. Remission (MADRS score ≤ 10) rates at week 4 were 38.46% and 16.00% in the active and sham group, respectively.

**Figure 2:**
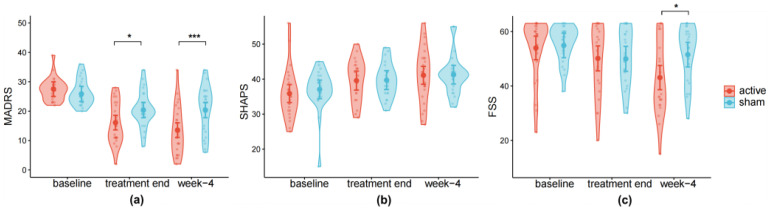
The clinical outcomes. Line charts of (**a**) MADRS, (**b**) Snaith-Hamilton Pleasure Scale (SHAPS), and (**c**) Fatigue Severity Scale (FSS) scores of participants in active and sham groups assessed at baseline (MADRS: N_active_ = 26, N_sham_ = 25; SHAPS: N_active_ = 25, N_sham_ = 24; FSS: N_active_ = 25, N_sham_ = 24), the last day of treatment (SHAPS: N_active_ = 21, N_sham_ = 23; FSS: N_active_ = 20, N_sham_ = 23), and 4 weeks after treatment (SHAPS: N_active_ = 21, N_sham_ = 23; FSS: N_active_ = 20, N_sham_ = 23). Dots indicate estimated marginal means, and error bars represent 95% confidence intervals (CI) computed from the model’s standard errors. Significance: * *p* < 0.05; *** *p* < 0.001.

For SHAPS, the main effect of time was significant (*F*_(2,90)_ = 12.156, *p* < 0.001, *η*^2^ = 0.214), but no significant main effect of group (*p* = 0.741) and interaction between time and group (*p* = 0.840) were found, suggesting comparable improvement across groups (Figure 2b).

For FSS, the model showed a significant main effect of time (*F*_(2,90)_ = 11.335, *p* < 0.001, *η*^2^ = 0.203), but no main effect of group (*p* = 0.259). Significant interaction between time and group was also observed (*F*_(2,90)_ = 4.626, *p* = 0.012, *η*^2^ = 0.094), scores in the active group were lower than the sham group (*β* = −8.434, SE = 3.213, *t*_(84)_ = −2.625, *p* = 0.010, 95% CI [−14.83, −2.03], *d* = −0.572; Figure 2c). These results showed a robust decrease in FSS scores over time and greater improvement in the treatment group, indicating a relatively delayed onset of rTMS effect on fatigue in MDD.

### 3.2. Affect Rating Task

#### 3.2.1. Analysis of Arousal and Valence Ratings

Linear mixed-effects models were fitted on ART data to assess arousal and valence ratings as a function of image category (positive, negative, neutral), time (baseline, treatment end, week 4), and group (active vs. sham). Separate models were estimated for arousal and valence ratings.

For valence ratings, the model revealed a significant main effect of image category (*F*_(2, 387)_ = 552.978, *p* < 0.001, *η*^2^ = 0.747), whereas the main and interaction effects involving time and group did not reach significance (all *ps.* > 0.110). Post hoc comparisons showed that, negative images were rated lower than both neutral (*β* = −1.519, SE = 0.254, *t*_(374)_ = −5.990, *p* < 0.001, 95% CI [−2.02, −1.02], *d* = −0.619) and positive images (*β* = −3.512, SE = 0.254, *t*_(374)_ = −13.806, *p* < 0.001, 95% CI [−4.01, −3.01], *d* = −1.430). Similarly, ratings of neutral images were lower than positive ones (*β* = −1.988, SE = 0.254, *t*_(374)_ = −7.818, *p* < 0.001, 95% CI [−2.49, −1.49], *d* = −0.810). These findings suggest that individuals with MDD did not show evidence of impaired emotional valence estimation.

For arousal ratings, the model revealed significant main effects of time (*F*_(2, 387)_ = 5.999, *p* = 0.003, *η*^2^ = 0.030) and image category (*F*_(2, 386)_ = 90.795, *p* < 0.001, *η*^2^ = 0.320), but no main or interaction effects involving group were significant (all *ps.* > 0.056). Post hoc comparisons indicated that negative images elicited higher arousal ratings than neutral images (*β* = −1.519, SE = 0.320, *t*_(386)_ = −4.750, *p* < 0.001, 95% CI [−2.15, −0.89], *d* = −0.483), but there was no significant difference for positive images (*p* = 0.199). In addition, at week 4, arousal ratings were significantly higher than baseline (*β* = 0.679, SE = 0.323, *t*_(387)_ = 2.099, *p* = 0.036, 95% CI [0.05, 1.31], *d* = 0.215). In sum, participants showed a normal pattern of differentiation between neutral and emotional (positive or negative) stimuli in terms of arousal, and their ability to elicit emotional arousal has increased.

#### 3.2.2. Correlations Between Baseline Ratings and Clinical Scales

**Figure 3:**
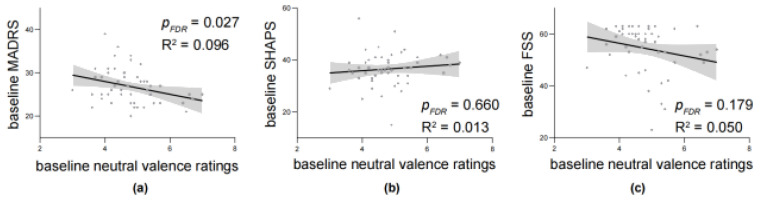
The correlation between baseline image ratings and baseline clinical scales. The correlation between baseline neutral valence ratings and baseline (**a**) Montgomery–Åsberg Depression Rating Scale (MADRS), (**b**) Snaith-Hamilton Pleasure Scale, (SHAPS) and (**c**) Fatigue Severity Scale (FSS). Dots are represented as individual data. Shadows represent 95% CI. *p*-values adjusted for multiple comparisons using FDR correction.

One interesting finding from the previous literature suggested that the affective processing towards neutral images may reflect the severity of depression (Leppänen et al., 2004). Thus, here we explored the correlation between baseline ratings of neutral images and clinical scales. Results showed that valence ratings of neutral images were negatively correlated with MADRS (N = 51, r = −0.310, R^2^ = 0.096, *p* = 0.027; Figure 3a) but not with SHAPS (N = 49, r = 0.112, R^2^ = 0.013, *p* = 0.660; Figure 3b) or FSS (N = 49, r = −0.223, R^2^ = 0.050, *p* = 0.179; Figure 3c).

#### 3.2.3. Predictive Effects of Emotional Arousal Change on Symptom Improvement

Since one main purpose of the current study is to investigate whether ART could serve as a predictor for rTMS efficacy, we then conducted linear regression models testing the association between ART changes and symptom improvements at week 4 in the active group (N = 26).

**Figure 4:**
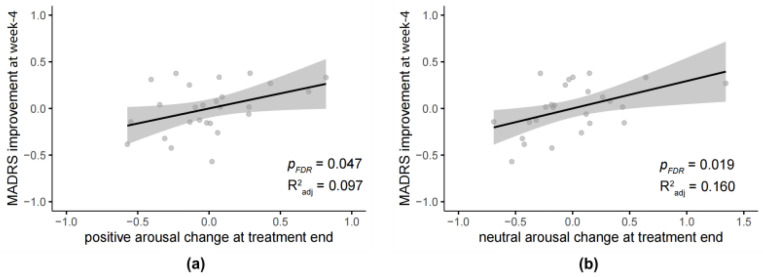
Regression of arousal changes at treatment end and MADRS improvement at week 4 in the active group. (**a**) Positive arousal change; (**b**) Neutral arousal change. Dots are represented as individual data. Shadows represent 95% CI. *p*-values adjusted for multiple comparisons using FDR correction; R^2^ indicates the adjusted coefficient of determination.

The model showed that, in the active group, the positive arousal change (F_(1,22)_ = 4.414, *β* = 0.428, *p* = 0.047, adjusted R^2^ = 0.097, 95% CI [0.00, 0.67]; Figure 4a) and neutral arousal change (F_(1,22)_ = 6.412, *β* = 0.483, *p* = 0.019, adjusted R^2^ = 0.160, 95% CI [0.05, 0.54]; Figure 4b) at treatment end could significantly positively predict the MADRS improvement at week-4. It means that participants in the active group who showed a larger increase in positive and neutral arousal change at treatment end exhibited greater reductions in depressive symptoms at week 4.

Then, multiple linear regression models including both active and sham groups with an interaction term were conducted to test whether such predictive effects are treatment-specific. Arousal change variables were mean-centered before entering the regression analysis. The models showed that, for positive arousal change, the interaction between group and arousal change was significant (*β* = −0.338, SE = 0.118, *t*_(47)_ = −2.877, *p* = 0.009, 95% CI [−0.57, −0.11], *d* = 0.840); and for neutral arousal change, the interaction was also significant (*β* = −0.230, SE = 0.078, *t*_(47)_ = −2.965, *p* = 0.006, 95% CI [−0.39, −0.07], *d* = 0.866). This indicates that the predictive effects are markedly specific to the active group.

The analysis above showed how emotional arousal predicts the improvement of MADRS. When addressing the SHAPS, while the participants exhibited some symptom relief, such improvement did not show between-group differences in our clinical analysis. Hence, it would be inappropriate to retain the improvement rate as the dependent variable. In this context, we next conducted linear regressions between these emotional predictors and week 4 anhedonia symptoms (SHAPS scores) in the active group (N = 25) to assess whether emotional changes serve as predictors of anhedonia symptoms after treatment. All results were nonsignificant (all *ps.* > 0.10). For FSS, though the improvement exhibited a significant difference between groups at week 4, none of the emotional predictors showed any significant association with the improvement in the active group (N = 25; all *ps.* > 0.10).

## 4. Discussion

In this study, we applied the accelerated dual-site rTMS protocol (CamFAST-P: Cambridge Fudan Accelerated Sequential TMS–Pudong) to MDD patients, which targeted dlPFC and dmPFC, with four treatments per day over 4 consecutive days. An affective rating task was conducted at different time points to explore the emotion processing function influenced by the treatment. We find that baseline valence ratings for neutral images are correlated with MADRS, but not with SHAPS or FSS. Intriguingly, changes in positive and neutral arousal at treatment end (day 4) are correlated with MADRS improvements at week 4 follow-up. Our results highlight the relationship between emotion processing and treatment efficacy and its role as a potential predictor following the accelerated dlPFC-dmPFC dual-site rTMS treatment.

Our results demonstrate that lower emotional valence of neutral images at baseline is associated with greater MDD symptom severity, supporting the theory that patients with depression exhibit strong negative biases (Gollan et al., 2008). Several studies have reported impaired recognition of neutral facial affect in depression, and that individuals with depression are more likely to misattribute neutral stimuli towards negative valence (Csukly et al., 2009; Leppänen et al., 2004; Maniglio et al., 2014). Cognitive theories of depression establish negative bias as a core pathogenic mechanism. Individuals with depression exhibit increased elaboration of negative information, impaired disengagement from negative material, and deficits in cognitive control when processing negative stimuli (Gotlib & Joormann, 2010), which posits its importance in understanding the pathology of depression. Neuroimaging studies have shown that functional connectivity patterns of negative stimuli are more likely to involve fronto-limbic connectivity, while neutral stimuli may exhibit distinct temporal-spatial signatures (Park et al., 2015; Wu et al., 2025). Previous literature has also reported negative bias in positive or negative stimuli (Bourke et al., 2010; Milders et al., 2010), which has not been found in the current experiment. A recent study has shown lower accuracy in MDD patients with more severe depressive symptoms only for neutral body affect recognition but not for negative or positive stimuli (Grzelak et al., 2025), suggesting that the degree of negative bias to neutral stimuli is more likely to reflect depression severity, rather than the diagnosis of MDD. Our results highlight the importance of emotion processing towards neutral stimuli in depressive symptoms.

An interesting finding from our study is that an increase in positive and neutral arousal after rTMS treatment (day 4) is shown to be correlated with improvement of MADRS at week 4, while no such effect has been revealed for negative arousal. These results suggest that our rTMS treatment may enhance the diminished emotional reactivity of positive and neutral stimuli in depression, indicating preferential modulation of positive emotion processing during antidepressant treatment. This aligns with evidence showing that individuals with milder depressive symptoms exhibit greater attentional engagement with happy stimuli rather than sad stimuli (Lazarov et al., 2018). Pharmacotherapy also acts on positive emotion during the early stage of treatment (Geschwind et al., 2011). Emotions such as excitement are linked to a state of high positive arousal. In contrast, a lack of energy or lassitude is a common symptom of depression. An increase in positive arousal can boost motivation and engagement, thereby improving these symptoms and contributing to a better MADRS outcome (Cutler et al., 2023). Our results echo previous findings and provide further evidence revealing that arousal towards positive and neutral stimuli may serve as indicators for depressive symptoms, thereby illuminating the complex neural networks implicated in these multidimensional symptom domains.

Anhedonia, a hallmark symptom of major depressive disorder (MDD) and a risk factor for treatment resistance and relapse (Wu et al., 2025), is a focus of our investigation. Our results show no significant improvement of anhedonia after our rTMS treatment, consistent with meta-analyses suggesting that rTMS lacks specific efficacy for this symptom (Li et al., 2025). This is clinically significant given that anhedonia predicts poor prognosis and heightened treatment resistance in MDD (Wong et al., 2024). Neuroimaging studies further indicate that anhedonia correlates with reward circuitry deficits (Höflich et al., 2019), suggesting that it may represent a distinct therapeutic target. Notably, anhedonia transcends diagnostic boundaries, manifesting in diverse psychiatric disorders, including schizophrenia, bipolar disorder, and post-traumatic stress disorder, etc., often persisting as a residual symptom (Lambert et al., 2018; Vinograd et al., 2022; Whitton & Pizzagalli, 2022). We highlight anhedonia as a cross-diagnostic construct in neuropsychiatric disorders, which may have a distinct neural circuitry. Future research into its neurobiological underpinnings may elucidate shared pathogenic mechanisms across seemingly disparate psychiatric conditions.

The 20 Hz rTMS protocol used in our study demonstrates favorable safety and tolerability profiles (Miron et al., 2019). Compared to the intermittent theta-burst stimulation (iTBS) used in Stanford Neuromodulation Therapy (SNT), a key advantage of this protocol is its capacity to deliver 1200 pulses within a 3 min session, doubling the pulse count of equivalent-duration iTBS. Neurophysiological and fMRI evidence indicate that 20 Hz stimulation may have a more consistent excitatory effect than 1 Hz, 10 Hz, or iTBS (Eldaief et al., 2011), potentially attributable to its duty cycle of 2 s on, 4 s off (Cash et al., 2017). Although head-to-head comparisons report comparable efficacy between the 20 Hz rTMS and iTBS protocol (André-Obadia et al., 2021), further investigation is warranted to reconcile the physiological superiority of higher pulse density with clinical outcomes.

There are several limitations in this study. First, the ART used in the study tests subjective feelings to emotional images. To avoid individual bias, we have applied the IAPS, which is a validated and the most frequently used database in emotion research. Development of objective paradigms, including behavior tasks and physiology measurements such as skin conductance, is required in studying emotion processing (Dritschel & Agren, 2023). Second, the current study only focuses on emotion processing, while depression is a complex psychiatric disorder characterized by abnormality in different dimensions of emotion cognition, including emotion recognition, processing, and regulation. Further studies are needed to systematically investigate the relationship between different aspects of emotion and antidepressant treatment of MDD. Third, the relatively small sample size may limit the interpretation of findings and reduce confidence in the results. To address this, future studies should prioritize larger, multicenter designs and replication in independent cohorts. Furthermore, incorporating multimodal predictors, including behavioral, neuroimaging, and physiological markers, combined with mediation analyses, could elucidate rTMS mechanisms more comprehensively.

In summary, the current study supports the negative bias theory in MDD. Our findings provide evidence showing the correlation between emotional arousal and clinical outcomes in MDD patients following the accelerated sequential dual-site rTMS treatment, suggesting its potential effect on MDD prognosis. Further investigation into the rTMS mechanism is warranted, given its clinical benefits in psychiatric disorders.

## Figures and Tables

**Table 1:** Demographic data.

	Active (*n* = 26)	Sham (*n* = 25)	Statistics	
			*t*/*χ*^2^	*p*
Sex (female/male)	12/14	18/7	3.515	0.061
Age (years, mean ± SD)	31.46 ± 6.94	32.24 ± 11.11	−0.301	0.764
Education (year, mean ± SD)	14.83 ± 3.56	14.88 ± 3.75	−0.520	0.959
Duration (month, mean ± SD)	47.81 ± 40.63	67.64 ± 70.01	−1.243	0.220
Episodes (mean ± SD)	1.50 ± 0.71	1.36 ± 0.49	0.819	0.417
Baseline MADRS (mean ± SD)	27.42 ± 3.82	26.24 ± 4.31	1.038	0.304
Medication (on/off)	26/0	24/1	1.061	0.303
SSRIs (on/off)	16/10	19/6	1.238	0.266
SNRIs (on/off)	8/18	8/17	0.009	0.925
Augmentation (on/off)	5/21	3/22	0.504	0.478
Benzodiazepines (on/off)	2/24	0/25	2.002	0.157

MADRS, Montgomery–Asberg Depression Rating Scale; SSRIs, selective serotonin reuptake inhibitors; SNRIs, serotonin and norepinephrine reuptake inhibitors.

## Data Availability

All data reported in this paper will be shared by the lead contact upon request.

## Abbreviations

The following abbreviations are used in this manuscript:

rTMS	Repetitive Transcranial Magnetic Stimulation
dlPFC	Dorsolateral Prefrontal Cortex
dmPFC	Dorsomedial Prefrontal Cortex
ART	Affect Rating Task
MDD	Major Depressive Disorder
ECN	Executive Control Network
DMN	Default Mode Network
MADRS	Montgomery-Åsberg Depression Rating Scale
SHAPS	Hamilton Pleasure Scale
FSS	Fatigue Severity Scale

## Appendix A

Eligibility criteria included individuals aged 18 to 55 years with a primary diagnosis of major depressive disorder based on DSM-5 criteria, MADRS score ≥ 20, and documented treatment failure or intolerance to at least one antidepressant at an adequate dose and duration in the current episode. Participants were required to maintain stable medication regimens for at least 4 weeks before enrollment and throughout the study period. Exclusion criteria comprised psychotic symptoms (including schizophrenia, schizoaffective disorder, and severe depressive episodes with psychotic features), history of epilepsy, stroke, or closed head injury, other serious medical or neurological conditions, prior history of ECT/MECT/TMS treatment, presence of implanted metallic devices, claustrophobia, and pregnancy. In addition, withdrawal criteria included participant death, loss to follow-up, refusal to continue treatment and/or withdrawal of informed consent, investigator-initiated termination, occurrence of intolerable adverse events requiring discontinuation, severe protocol deviations, and meeting exclusion criteria that were newly discovered or confirmed during the study.

Sample size was calculated using G*Power 3.1 for an estimated effect size of Cohen’s d = 0.8 for the change in MADRS between active and sham groups. With the type I error rate of 5% (two-sided) and power of 80%, 26 participants are required.
